# RNA-binding protein AUF1 suppresses cellular senescence and glycolysis by targeting *PDP2* and *PGAM1* mRNAs

**DOI:** 10.18632/aging.206286

**Published:** 2025-07-24

**Authors:** Hyejin Mun, Chang Hoon Shin, Mercy Kim, Jeong Ho Chang, Je-Hyun Yoon

**Affiliations:** 1 Department of Oncology Science, College of Medicine, University of Oklahoma, Oklahoma City, OK 73104, USA; 2 Department of Biology Education, Kyungpook National University, Daegu 41566, Republic of Korea; 3 Department of Pathology, College of Medicine, University of Oklahoma, Oklahoma City, OK 73104, USA

**Keywords:** AUF1, MST1, senescence, glycolysis, aging

## Abstract

Signaling pathways and transcriptional regulation during cellular senescence have been investigated; however, energy metabolism is one of the understudied areas. Senescent cells secrete pro-inflammatory cytokines and release proteins and RNAs via exosomes that contribute to organismal aging. Although senescent fibroblasts in solid organs are in a low oxygen environment, these fibroblasts have more active glucose metabolism and consume more oxygen than proliferating ones. A critical gap in our knowledge is how senescent fibroblasts facilitate glucose metabolism and organismal aging by creating a distinct microenvironment. Our high throughput profiling of mRNAs and proteins from Human Diploid Fibroblasts (HDFs) revealed that the expression of pyruvate metabolic enzymes is inhibited by the anti-senescent RNA-binding protein (RBP) AUF1 (AU-binding Factor 1). Our studies revealed that AUF1 promotes the decay of mRNAs encoding two enzymes: PGAM1 (phosphoglycerate mutase 1), a glycolytic enzyme involved in the pyruvate synthetic pathway, and PDP2 (Pyruvate Dehydrogenase Phosphatase 2), which activates Pyruvate Dehydrogenase. We also demonstrated that AUF1 is phosphorylated by a Serine/Threonine kinase, MST1 (Mammalian Ste20-like kinase 1; encoded by STK4), resulting in the inactivation of AUF1, which leads to target mRNA stabilization and senescence. Overexpression of PGAM1 and PDP2 predicts an acceleration of pyruvate production and subsequent citrate metabolism, leading to cellular senescence and aging. Thus, our studies revealed regulatory mechanisms of glycolysis-driven cellular senescence by AUF1-mediated decay of *PGAM1* and *PDP2* mRNAs.

## INTRODUCTION

Senescence is a cellular process by which proliferating cells lose their ability to divide, grow, and proliferate [1, 2]. Cellular senescence can be triggered by an accumulation of chromosomal damage due to inaccurate DNA replication, DNA segregation, and DNA breakage/repair [1, 2]. Chromosomal damage that mainly occurs via continuous DNA replication, ionizing irradiation, oncogenic mutations, hormonal changes, and alcohol consumption subsequently leads to the generation of reactive oxygen species (ROS) [1–4]. In response to excessive chromosomal damage, most cells, including fibroblasts, cease further division in order to prevent tumor initiation and become senescent [5]. These cells also secrete various pro-inflammatory cytokines and extracellular vesicles that impact neighboring cells, which can drive paracrine-mediated senescence [6]. In order to secrete these molecules, senescent fibroblasts rely on using more glucose and oxygen than proliferating fibroblasts, even under hypoxic conditions [7]. Importantly, senescent fibroblasts prefer to convert pyruvate to Acetyl-CoA/citrate for acceleration of fatty acid synthesis and membrane trafficking [8]. It is notable that budding yeast grown under low-glucose media (0.05%) have a longer lifespan than those grown under normal glucose media (2%) [9].

In this study, we examined a current knowledge gap in this field: How do senescent fibroblasts accelerate pyruvate/Acetyl-CoA/citrate metabolism to facilitate pro-inflammatory cytokine secretion? We addressed the overarching challenge involving the post-transcriptional regulation of glucose metabolic enzymes that promote cellular senescence. We discovered that 1) two metabolic enzymes, PGAM1 and PDP2, are up-regulated in response to the replicative senescence (RS)-induced phosphorylation of AUF1 (AU-binding factor 1), an anti-senescent and anti-aging RNA-binding protein (RBP) [10–12], and 2) AUF1 phosphorylation is accelerated by the MST1 (Mammalian Ste20-like Kinase 1) Ser/Thr kinase [13]. These findings are novel and important since they support the hypothesis that the MST1-AUF1-PGAM1/PDP2 pathway exerts a critical function in modulating RS [14]. Activation of the Ser/Thr kinase MST1 by ROS mediates the specific phosphorylation of the RBP AUF1, resulting in the dissociation of AUF1 from *PGAM1* and *PDP2* mRNAs and their stabilization, which would enhance glucose metabolism. Facilitating glucose metabolism would mediate the synthesis of fatty acids as well as the secretion of pro-inflammatory cytokines [15, 16], thus leading to cellular senescence.

At a fundamental level, this study is unique because it examines the post-transcriptional regulatory mechanism(s) controlling glucose metabolism. Thus, the current study is of high significance and impact because it will lead to a better understanding of glucose metabolism and senescence in response to repeated DNA replication. This range of studies will allow us to determine the role of the MST1-AUF1-PGAM1/PDP2 pathway in senescence driven by glucose metabolism [17, 18].

## RESULTS

### AUF1 deficiency accelerates senescence by targeting glycolytic enzymes

**Figure 1:**
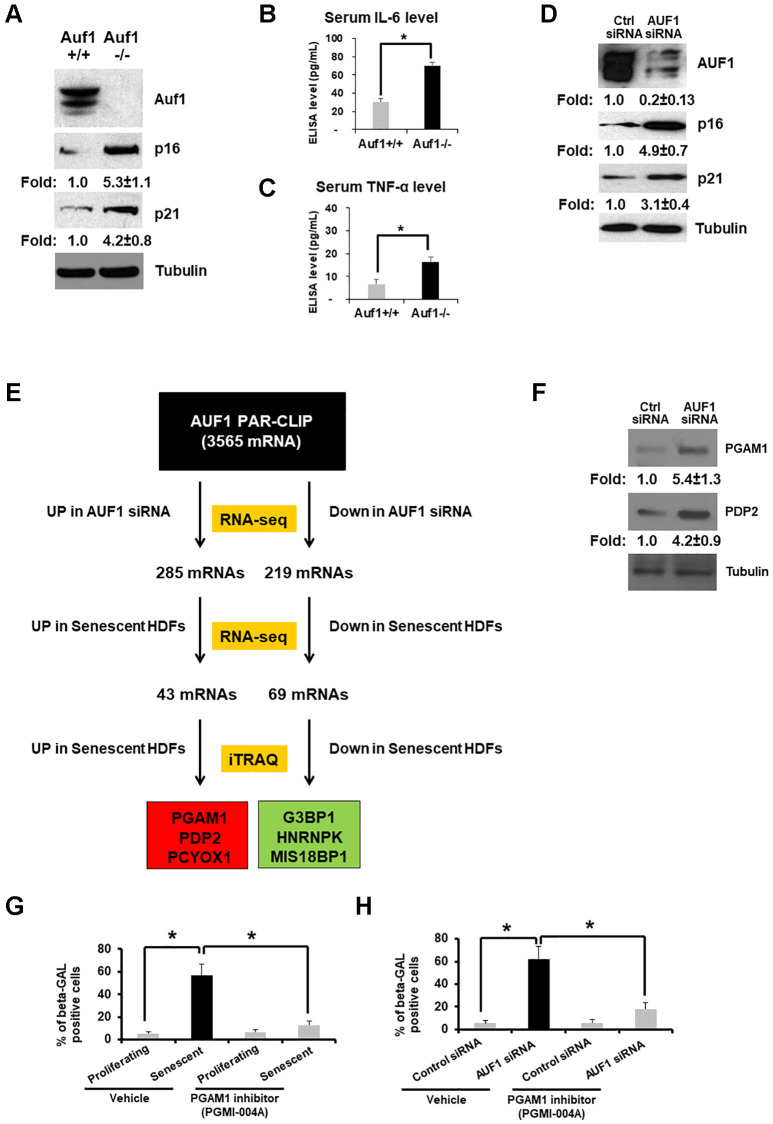
**AUF1 deficiency accelerates senescence by targeting glycolytic enzymes.** (**A**) Western blot analysis of Auf1, p16, p21, and Tubulin from primary lung fibroblasts of *Auf1*^+/+^ and *Auf1*^−/−^ mice. (**B**, **C**) Serum levels of IL-6 (**B**) and TNF-α (**C**) isolated from *Auf1*^+/+^ and ^−/−^ mice (*n* = 4, 23-week-old mice). The results shown in (**A**) represent data from four independent experiments. The graph in (**B**) and (**C**) is an average ± standard deviation (S.D.) of four mice (^*^p < 0.05). (**D**) Western blot analysis of AUF1, p16, p21, and Tubulin in lysates from proliferating HDFs transfected with control or AUF1 siRNA for 72 hours. (**E**) The pipeline for identifying mRNAs targeted by AUF1 for the regulation of mRNA and protein abundance in replicative senescence via direct binding of AUF1 with target mRNAs. (**F**) Western blot analysis of PGAM1, PDP2, and Tubulin from proliferating HDFs transfected with control or AUF1 siRNA. The data shown in (F) represent three independent experiments. (**G**, **H**) Number of β-GAL-positive fibroblasts in the presence or absence of a chemical inhibitor against PGAM1, PGMI-004A (20 μM), in proliferating or senescent fibroblasts (**G**) transfected with control or AUF1 siRNA (**H**). The graphs in (**G**) and (**H**) are an average ± S.D. of three independent experiments (^*^p < 0.001).

To understand the *in vivo* function of AUF1 (AU-binding factor 1) in senescence and aging, we created a conditional, fibroblast-specific AUF1 knockout model with the C57BL/6 background. The *Hnrnpd* gene that encodes AUF1 is located on murine chromosome 5 and contains nine exons. Our deletion of exons 3–7 resulted in a complete AUF1 loss-of-function by generating a frame shift from the downstream exon. In the targeting vector, LoxP sites flanked the CKO region. The constitutive KO allele, generated after Cre-mediated recombination in the germline (Figure 1A), has shown an increase in the levels of p16 and p21 which are known for cell cycle arrest [5]. Crossing LoxP mice with a fibroblast-specific CreER expressed under the control of the FSP promoter produced fibroblast-specific knockout mice from P8-P10 with the C57BL/6 background. By comparing the serum cytokine levels of both *Auf1^+/+^* and *Auf1*^−/−^ mice, we demonstrated that *Auf1* deficiency elevates serum levels of IL-6 and TNF-α (Figure 1B, 1C), which is consistent with observations in whole-body *Auf1*^−/−^ mice [17] and wild-type 12-month-old mice with exogenous AUF1 [12].

To study molecular mechanisms of how AUF1 suppresses aging processes, we established a human diploid fibroblast (HDF) model of senescence that mimics organismal aging. AUF1 is highly downregulated in replicative senescent (RS) fibroblasts (WI-38, PDL55) [19], and its depletion is sufficient to promote fibroblast senescence, as indicated by increased expression of p16 and p21 (Figure 1D), as well as genomic DNA fragmentation [19]. We previously observed that AUF1 depletion increased the Olive Tail Moment of HDFs in a single-cell gel electrophoresis assay (also called a Comet assay) [19]. These findings align with previous reports that AUF1 is a suppressor of RS [12, 17, 19].

To investigate whether AUF1 regulates senescence by modulating gene expression, we identified: 1) AUF1 target mRNAs by Photoactivatable Ribonucleoside-Enhanced Crosslinking and Immunoprecipitation (PAR-CLIP) [19]; 2) Differential expression of mRNAs in AUF1-silenced proliferating HDFs compared to control-silenced cells, as well as the comparison between proliferating and senescent HDFs, was assessed using high-throughput RNA sequencing (RNA-seq) [19]; and 3) proteins differentially expressed in senescent fibroblasts by Isobaric Tags for Relative and Absolute Quantitation (iTRAQ) and Mass Spectrometry (MS) (Figure 1E).

Among the 3,565 mRNAs targeted by AUF1, 285 were found to be overexpressed in AUF1-depleted HDFs, while 219 were underexpressed. A comparison of these mRNAs with those from senescent HDFs revealed 43 mRNAs that were up-regulated and 69 that were down-regulated in both AUF1-depleted and senescent HDFs. We then evaluated whether these selected genes consistently reflected changes in protein expression through iTRAQ analysis (Figure 1E). Our subsequent analysis identified the top 3 proteins that were either overexpressed or underexpressed in the context of AUF1-mediated cellular senescence: 1) PGAM1 (Phosphoglycerate Mutase 1 catalyzing 3-phosphoglycerate to 2-phosphoglycerate), 2) PDP2 (Phosphatase Catalytic Subunit 2 of Pyruvate Dehydrogenase catalyzing pyruvate to Acetyl-CoA), and PCYOX1 were up-regulated, while G3BP1, hnRNPK, and MIS18BP1 were down-regulated. We confirmed overexpression of PGAM1 and PDP2 in AUF1-depleted HDFs using western blot analysis (Figure 1F). Previously, we analyzed changes in *PDP2* mRNA stability and found that PDP2 mRNA stability increases after AUF1 depletion [20]. Treatment with a PGAM1 inhibitor decreased the number of senescent fibroblasts induced by repeated DNA replication in HDFs (Figure 1G) or AUF1 depletion in primary fibroblasts (Figure 1H).

### Senescent fibroblasts overexpress *PGAM1* and *PDP2* mRNAs to promote cellular senescence and glycolysis

**Figure 2:**
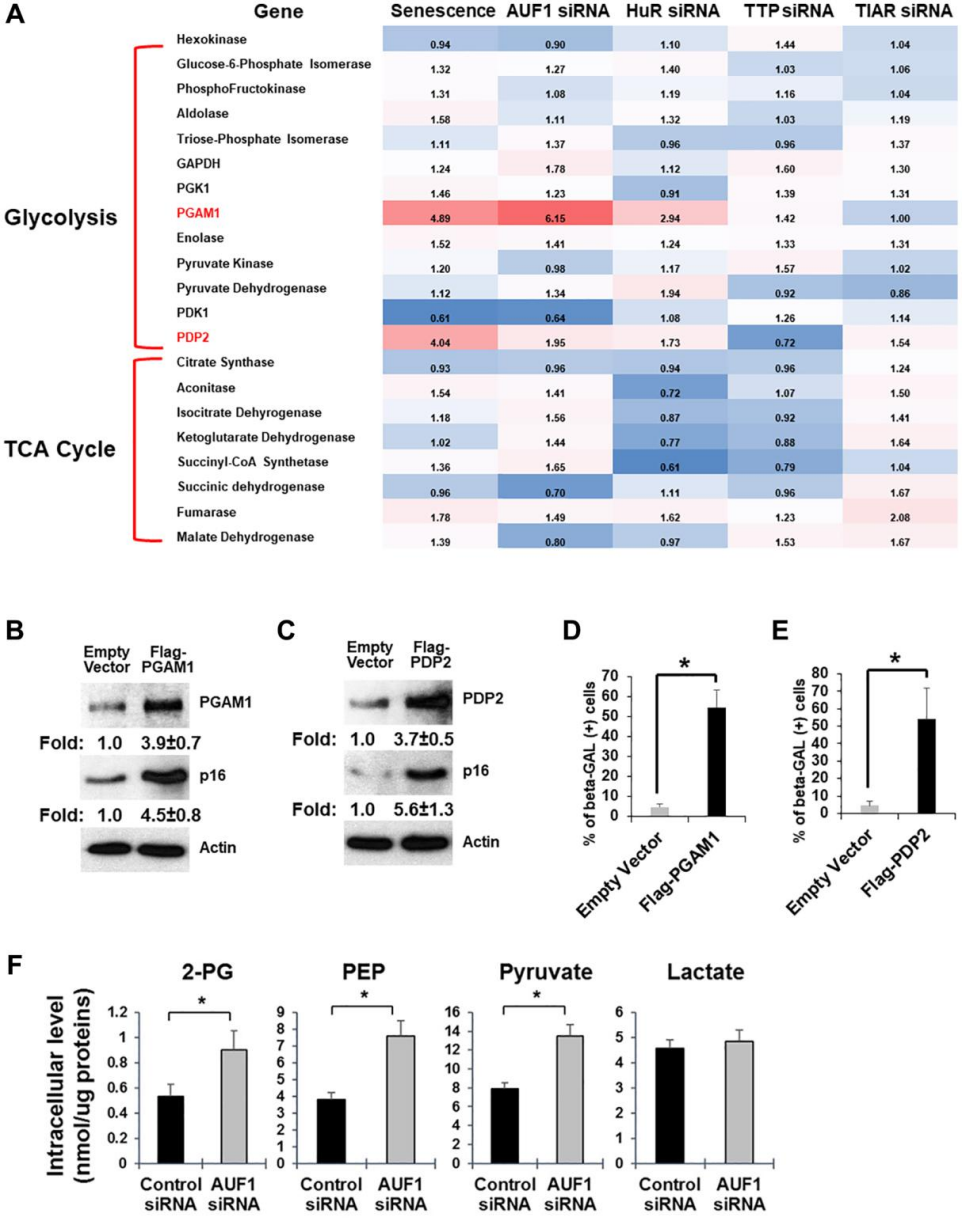
**Senescent fibroblasts overexpress *PGAM1* and *PDP2* mRNAs to promote cellular senescence and glycolysis.** (**A**) Fold changes in mRNAs encoding proteins involved in glycolysis and the TCA cycle. The comparison was performed in senescent HDFs and proliferating HDFs or proliferating HDFs transfected with siRNAs targeting AUF1, HuR, TTP, or TIAR and proliferating HDFs transfected with control siRNAs. (**B**, **C**) Western blot analysis of PGAM1, PDP2, p16, and Tubulin using cell lysates purified from proliferating HDFs transfected with cDNA plasmids of Flag-PGAM1 or PDP2, compared to an empty vector. The results in (**B**) and (**C**) represent three independent experiments. (D and E) Senescence-associated β-galactosidase assay using proliferating HDFs transfected with Flag-PGAM1 or PDP2 plasmids, compared to an empty vector. The graphs in (**D**) and (**E**) are averages ± standard deviations (S.D.) of three independent experiments (^*^p < 0.001). (**F**) Colorimetric measurement of a subset of glucose metabolites using cell lysates (for 2-PG, PEP, and pyruvate) or culture media (for lactate) from HDFs transfected with control or AUF1 siRNAs. The graphs are averages ± S.D. of four independent experiments (^*^p < 0.001).

Senescent fibroblasts expend more oxygen than proliferating ones [8, 21]. If senescence is regulated by glucose metabolism, there should be changes in the activity of metabolic enzymes and/or their expression levels [22]. Our total RNA-seq of proliferating and senescent HDFs (PDL15 vs. PDL55) has shown that a subset of glucose metabolic enzymes is overexpressed or underexpressed in senescent fibroblasts (Figure 2A). We also compared mRNA expression of these enzymes after the depletion of AUF1, HuR, TTP, or TIAR; all are RBPs whose silencing affects cellular senescence [17, 23–25]. Among these RBPs, AUF1 depletion by siRNA exhibits the most similar profile of gene expression when compared to the overexpression of *PGAM1* and *PDP2* mRNAs in senescent fibroblasts. HuR depletion also increased the levels of *PGAM1* and *PDP2* mRNAs to a similar degree as senescent HDFs and AUF1-depleted fibroblasts. Overexpression of PGAM1 or PDP2 by exogenous plasmids was sufficient to increase the level of p16, a senescence marker protein, in proliferating HDFs (Figure 2B, 2C). We also observed an increase in beta-galactosidase (β-gal)-positive HDFs after overexpression of PGAM1 or PDP2 (Figure 2D, 2E) [16]. This suggests the existence of alternative pathways that regulate glucose metabolism at post-transcriptional levels.

Overexpression of PGAM1 and PDP2 in AUF1-depleted HDFs predicts a change in glucose metabolism in the conversion of 3-phosphoglycerate (3-PG) to 2-phosphoglycerate (2-PG) and in pyruvate conversion to Acetyl-CoA. Our measurement of glucose metabolites has shown that the levels of 2-PG, PEP, and pyruvate increase, while the level of lactate remains unchanged (Figure 2F). Our data indicates that AUF1 may function as a repressor of glucose metabolism, possibly by restricting the expression of PGAM1 and PDP2.

### MST1-AUF1 pathway inhibits mitochondrial function and mTOR activity to promote senescence

**Figure 3:**
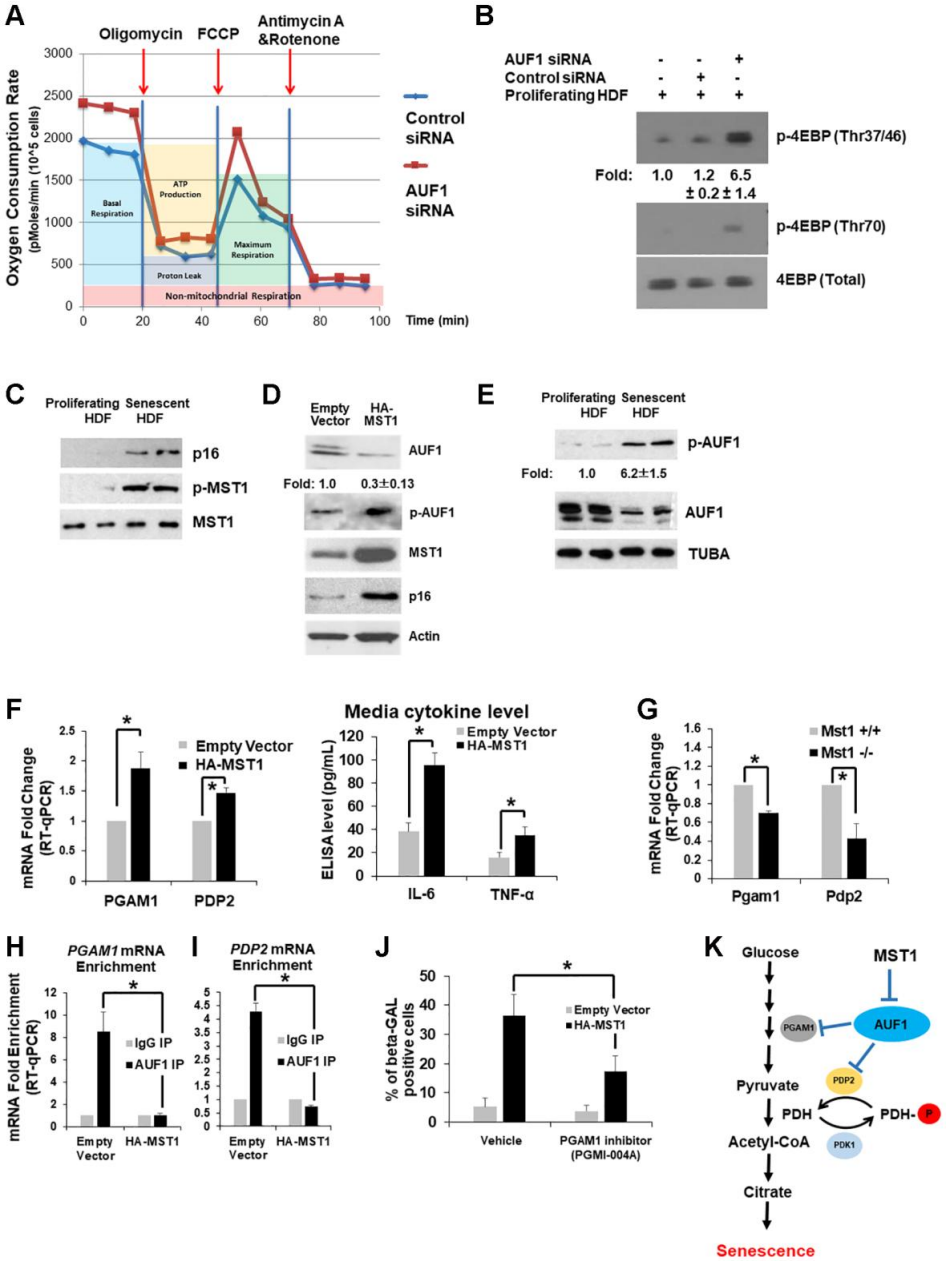
**MST1-AUF1 pathway inhibits mitochondrial function and mTOR activity to promote senescence.** (**A**) Measurement of the oxygen consumption rate using proliferating HDFs transfected with control or AUF1 siRNAs after treatment with oligomycin, FCCP, and Antimycin A and Rotenone over a given time course. (**B**) Western blot analysis of phosphorylated 4EBP on Thr37/46 and Thr70, indicating mTOR activity, a critical regulator of glucose metabolism and senescence. The results in (**A**) and (**B**) represent three independent experiments. (**C**) Western blot analysis of phosphorylated MST1 on Thr183 and total MST1 in proliferating and senescent HDFs. (**D**) Western blot analysis of AUF1, p-AUF1, MST1, and p16 in proliferating HDFs transfected with an empty vector or the cDNA plasmid of HA-tagged MST1. (**E**) Western blot analysis showing the levels of p-AUF1 and AUF1 in cell lysates from proliferating and senescent HDFs. The results in (**B**–**E**) represent three independent experiments. (**F**) RT-qPCR levels of *PGAM1* and *PDP2* mRNAs from total RNAs purified from proliferating fibroblasts transfected with an empty vector or the cDNA plasmid of HA-tagged MST1 (*left*). Media levels of IL-6 and TNF-α isolated from proliferating fibroblasts transfected with an empty vector or the cDNA plasmid of HA-tagged MST1 (*right*). (**G**) RT-qPCR levels of *Pgam1* and *Pdp2* mRNAs from total RNAs purified from Mst1^+/+^ and Mst1^−/−^ mouse lung fibroblasts. (**H**, **I**) RT-qPCR levels of co-purifying *PGAM1* (**H**) and *PDP2* mRNAs (**I**) from immunoprecipitated AUF1 in HDFs transfected with an empty vector or HA-tagged MST1, compared to IgG control precipitates. The graphs are averages ± standard deviations (S.D.) of four independent experiments (^*^p < 0.001). (**J**) Number of β-GAL-positive fibroblasts in the presence or absence of a chemical inhibitor against PGAM1, PGMI-004A (20 μM), in proliferating fibroblasts transfected with an empty vector or the cDNA plasmid of HA-tagged MST1. The graphs in (**J**) are an average ± S.D. of three independent experiments (^*^p < 0.001). (**K**) Schematic representation of the MST1-AUF1-PDP2/PGAM1 pathway in the regulation of glycolysis and senescence.

Because glucose metabolism is directly connected to mitochondrial function and the mTOR pathway, we also assessed the oxygen consumption rate (OCR) after AUF1 depletion in proliferating fibroblasts. In a process similar to one initiated by senescent fibroblasts [8], AUF1 silencing increased the basal and maximum respiration of cells following treatment with either oligomycin or FCCP, which are inhibitors of mitochondrial function (Figure 3A). Accelerated glucose metabolism and oxygen consumption were also reflected by an increase in mTOR activity, as represented by the phosphorylation of its substrate, 4EBP, at multiple residues in AUF1-depleted fibroblasts (Figure 3B). This finding indicates that AUF1 is critical for the maintenance of both mitochondrial respiration and the mTOR pathway.

Our previous studies revealed that Ste20 in budding yeast phosphorylates the decapping enzyme 2 (Dcp2) upon oxidative stress [26]. We also observed that MST1 phosphorylates the eukaryotic mRNA translation initiation factor 4E (eIF4E) in human and mice [27]. MST1 binds ROS-sensing protein thioredoxin directly [28], and the kinase activity of MST1 is elevated by accumulation of. To test if MST1 mediates ROS-induced senescence via inactivation of AUF1 [3, 4], we investigated the phosphorylation status of MST1 at Thr183 in whole-cell lysates purified from proliferating and senescent fibroblasts (PDL15 and PDL55). We found that MST1 kinase activity was escalated (p-MST1) in senescent fibroblasts (Figure 3C). Our findings demonstrate that MST1 mediates signaling pathways regulating the expression of AUF1.

As examined in senescent fibroblasts with more active MST1 (Figure 3C), overexpression of MST1 in proliferating fibroblasts increases the level of p16 and decreases the level of AUF1; we observed a ~30% reduction of AUF1 but not complete depletion with AUF1 phosphorylation (Figure 3D). We hypothesized that AUF1 could be a substrate of MST1, and AUF1 phosphorylation might affect its expression level in senescent fibroblasts. To test this, we utilized active recombinant MST1 and AUF1 proteins from E. coli and performed a phosphorylation reaction *in vitro* between AUF1 and MST1. Mass Spectrometry revealed that the MST1 phosphorylates on AUF1 resides at Thr127 (Figure 3E) as we identified from alcohol-induced intestinal injury [35]. Importantly, analysis of AUF1 crystal structure exhibited that the Thr127 residue is located within the RNA-binding pocket of AUF1 [29]. Our western blot analysis also revealed that AUF1 phosphorylation at Thr127 (p-AUF1) increased in senescent HDFs (Figure 3E). Taken together, these results suggest that in senescent fibroblasts, AUF1 phosphorylation is induced at residue Thr127 by MST1. Overexpression of MST1 in proliferating fibroblasts increases the expression of *PGAM1* and *PDP2* mRNA as well as media levels of IL6 and TNF-α (Figure 3F). Lung fibroblasts isolated from *Mst1^+/+^* and *Mst1*^−/−^ (Jackson Laboratory) mice showed that *Pgam1* and *Pdp2* mRNAs are less abundant in the MST1-deficient fibroblasts (Figure 3G). Overexpression of HA-MST1 in HDFs decreased the enrichment of *PGAM1* and *PDP2* mRNA co-purified with AUF1 from HDF lysates (Figure 3H, 3I). Moreover, PGAM1 inhibitor suppresses senescence induction after MST1 overexpression (Figure 3J). These results suggest that in the process of cellular senescence, MST1 phosphorylates AUF1, leading to its inactivation and the stabilization of *PGAM1* and *PDP2* mRNA.

## DISCUSSION

Extensive research has focused on identifying marker genes, such as senescence-associated secretory phenotypes (SASPs) [1, 2, 6, 16], related to cellular senescence; however, the alterations in cellular metabolism within senescent cells remain inadequately understood. This study investigates the role of AUF1 (AU-binding factor 1) in cellular senescence and glucose metabolism. We observed a reduction in AUF1 levels in replicative senescent (RS) fibroblasts [19] (Figure 3E). Moreover, the depletion of AUF1 in mouse models and proliferating fibroblasts resulted in an increase in senescence, characterized by several changes in senescence-associated proteins (Figure 1A–1D). Through the analysis of Photoactivatable Ribonucleoside-Enhanced Crosslinking and Immunoprecipitation (PAR-CLIP) combined with RNA-sequencing (RNA-seq) and Isobaric Tags for Relative and Absolute Quantitation (iTRAQ) Mass spectrometry (MS) data, we identified a refined set of up or down-regulated genes in senescent fibroblasts as well as in AUF1-silenced proliferating cells (Figure 1E). There are three down-regulated genes identified in this study: G3BP1, hnRNPK, and MIS18BP1. Omer et al. demonstrated that G3BP1 plays a critical role in cellular senescence, particularly in regulating the senescence-associated secretory phenotype (SASP). During senescence, G3BP1 facilitates the association of cyclic GMP-AMP synthase (cGAS) with cytosolic chromatin fragments, activating the NF-κB and STAT3 pathways, which in turn leads to the expression and secretion of SASP factors. Notably, the depletion or inhibition of G3BP1 prevents SASP expression without affecting the commitment of cells to senescence. This indicates that while cells still enter senescence, they do not secrete the factors typically associated with tumor growth and inflammation [30]. Additionally, Shin et al. reported that hnRNPK is crucial for cellular senescence by regulating CDC20, a key protein involved in cell cycle progression. During senescence, levels of hnRNPK decline, resulting in reduced transcription and stability of CDC20 mRNA, which subsequently decreases CDC20 production and contributes to cell cycle arrest [31]. Furthermore, while MIS18BP1 is primarily recognized for its role in centromere assembly and chromosome segregation, its involvement in senescence remains less characterized. However, studies suggest that MIS18BP1 may influence cell cycle regulation and apoptosis by modulating p53, potentially impacting senescence-related pathways [32]. On the other hand, among the up-regulated genes, PGAM1 and PDP2, both involved in glucose metabolism, were significantly up-regulated following AUF1 silencing, and the overexpression of either PGAM1 or PDP2 promoted senescent phenotypes (Figure 2). Mechanistically, the activation of MST1 in senescent cells inhibits the binding ability of AUF1 to PGAM1 and PDP2, which subsequently upregulates the levels of PDP2 and PGAM1, enhancing glycolysis in senescent cells (Figure 3).

### Phosphorylation of AUF1 mediated by MST1 activation in mRNA decay

MST1 phosphorylation at threonine 183 (Thr183), induced by oxidative stress, regulates cell growth and apoptosis through the Hippo pathway [33]. This phosphorylation is critical in modulating the interaction between RNA-binding proteins (RBPs) and their target RNAs, potentially leading to alterations in gene expression. In previous research, Ste20 was identified as a kinase that phosphorylates decapping enzyme 2 (Dcp2) at serine 137 (Ser137) in response to oxidative stress, thereby enhancing Dcp2’s role in mRNA decay in yeast [26]. More recently, MST1 has been shown to phosphorylate eIF4E at threonine 55 (Thr55), which decreases its binding affinity for eIF4E target mRNAs (*eIF2α*, *CCT2*, and *eEF2*), inhibiting their translation while promoting the translation of specific long non-coding RNAs (*linc00689* and *linc00854*) [27]. The most recent study demonstrated that during adipocyte differentiation, the activation of MST1 phosphorylates RBP, Me31b stabilizes its target *Glucagon* mRNA, thereby promoting lipolysis in adipocytes [34]. Additionally, the activation of MST1 can result in the phosphorylation of AUF1 at threonine 127 (Thr127) during alcohol-induced damage, which diminishes the interaction between CYP2E1-targeting miRNAs and AUF1, leading to the degradation of these miRNAs and an increase in CYP2E1 levels [35]. In alignment with earlier findings, our study demonstrates that elevated phosphorylation of MST1 in RS cells enhances AUF1 phosphorylation and reduces AUF1 enrichment on *PGAM1* and *PDP2* mRNAs, resulting in increased expression of these mRNAs (Figure 3E, 3F, 3H– 3I). Further research is necessary to elucidate the detailed mechanisms by which MST1-mediated AUF1 phosphorylation regulates these mRNAs.

### The role of AUF1 in glycolysis

AUF1, also known as hnRNP D, plays a critical role in cellular regulation, demonstrating its multifaceted involvement in essential biological processes [36, 37]. Previous research has established that AUF1 is vital for telomere maintenance, where it activates telomerase transcription, preserves telomere integrity, and safeguards genomic stability by preventing DNA damage, thereby delaying cellular senescence and organismal aging [38, 39]. Furthermore, AUF1 acts as a suppressor of inflammation by destabilizing inflammatory cytokine mRNAs, contributing to the regulation of aging [38, 39]. Mechanistically, AUF1 intricately interacts with miRNA pathways. By enhancing miRNA, let-7b loading onto Argonaute 2 (AGO2), AUF1 facilitates gene silencing and mRNA decay, thus influencing post-transcriptional gene regulation [20, 40]. Its selective binding to U-/GU-rich RNA sequences enables AUF1 to modulate the stability, decay, or translation of both coding and non-coding RNAs [19]. Notably, AUF1 modulates the long non-coding RNA NEAT1; it binds to NEAT1 and promotes its degradation, resulting in reduced steady-state levels of NEAT1 [19]. Although research on the role of AUF1 in glucose metabolism is limited, a recent study has underscored the functional role of NEAT1 in glycolysis related to breast cancer progression [41]. Elevated levels of NEAT1 in breast cancer enhance glycolysis by scaffolding key glycolytic enzymes, including PGK1, PGAM1, and ENO1, concurrently within the same cellular context, thereby promoting breast cancer progression and metabolism [41]. Given that NEAT1 is a target of AUF1 and plays crucial roles in glycolysis, it is plausible that AUF1 may also be involved in glycolytic processes. One piece of evidence supporting this notion is that AUF1 facilitates the interaction between let-7b and AGO2, resulting in the repression of *PDP2* mRNA, which encodes the pyruvate dehydrogenase phosphatase catalytic subunit 2 [20]. This observation, consistent with our current study, suggests that AUF1 may play a role in influencing glucose metabolism (Figure 2C).

### The role of glucose metabolism in cellular senescence

Senescence is a dynamic biological process characterized by a gradual decline in cellular function, often leading to aging and age-related diseases [42]. This process is marked by cell cycle arrest, metabolic alterations, and the secretion of pro-inflammatory molecules [43–46]. Glucose metabolism plays a crucial role in senescence by providing energy and intermediates for cellular processes [47, 48]. Notable alterations in glucose metabolism, such as increased glycolysis and modifications in glucose transporter activity, are frequently observed in senescent cells [49–52]. For instance, the expression of the glucose transporter GLUT1 is elevated in senescent cells, resulting in enhanced glucose uptake and increased glycolytic activity [49]. Additionally, key glycolytic enzymes, including hexokinases (HKs) and lactate dehydrogenase A (LDHA) [51, 52], are upregulated in senescent cells, further driving glycolysis and producing pyruvate, which subsequently enters the tricarboxylic acid (TCA) cycle [52]. Consistent with previous studies, we observed that elevating the levels of other glycolytic enzymes, such as PGAM1 and PDP2, in AUF1-depleted cells enhances glucose metabolism through increasing production of pyruvate in those senescent cells (Figure 2B, 2C, 2F). Furthermore, a recent study demonstrated that senescent vascular endothelial cells undergo a shift towards increased oxidative glucose metabolism [47]. This transition is facilitated by reduced expression of pyruvate dehydrogenase kinase (PDHK), which enhances the activity of pyruvate dehydrogenase and promotes oxidative pathways. Consequently, senescent cells exhibit an elevated oxygen consumption rate (OCR) [47]. This finding corroborates our results, indicating that senescent cells triggered by AUF1 silencing display an increased OCR, potentially attributable to the upregulation of one of the pyruvate dehydrogenases, PDP2 (Figure 3A). These insights underscore the significant impact of glucose metabolism on the regulation of senescence and highlight potential interventions for aging and age-related diseases.

### The MST1-AUF1-PDP2/PGAM1 pathway: A potential target for senescence regulation and senotherapeutics

Our findings demonstrate that MST1 phosphorylation enhances the expression of PDP2 and PGAM1 by reducing AUF1 binding affinity to their mRNA, thereby promoting glucose metabolism in RS fibroblasts (Figure 3K). This identifies the MST1-AUF1-PDP2/PGAM1 as a potentially unique senescence-associated pathway. Given the dynamics of cellular senescence, employing diverse triggers to induce senescence at various time points could strengthen the evidence that this pathway is intricately linked to DNA damage responses [31, 42, 53]. Exploring this approach would help confirm the robustness of our findings. Since MST1 phosphorylation and PDP2/PGAM1 levels are elevated during senescence, current inhibitors targeting these molecules hold promise for mitigating or potentially reversing senescence [54]. Combining these inhibitors with senolytic drugs could result in a synergistic effect, selectively eliminating senescent cells and enhancing therapeutic outcomes [55]. Furthermore, glucose metabolism in senescent cells is tightly linked to SASP factor secretion [46, 48]. Modulating this pathway could provide a novel strategy for senotherapy, potentially reshaping senescence-associated phenotypes [56]. Altogether, the post-transcriptional regulation of PDP2 and PGAM1 in senescence offers a compelling avenue for the development of innovative senotherapeutics aimed at addressing aging and age-related diseases.

## MATERIALS AND METHODS

### Cell culture, transfection, short hairpin RNAs, and plasmids

Human diploid fibroblast (WI-38), Mouse Embryonic Fibroblast (MEF) cells were cultured in DMEM (Invitrogen, Waltham, MA, USA) supplemented with 10% (v/v) FBS and antibiotics. Using Lipofectamine 2000 (Invitrogen) or PEI (polyethylenimine), the cells were then transfected with either siControl (UUCUCCGAACGUGUCACGUdTdT, targeting GFP), or siAUF1 (AAGAUCCUAUCACAGGGCGAUdTdT). MST1 plasmids are from Dr. Eui-Ju Choi. PGAM1 and PDP2 plasmids were purchased from Addgene (Watertown, MA, USA). Cells were analyzed for RT-qPCR, western blot, and extracellular vesicles purification 48 hours after transfection.

### RNP analysis

Immunoprecipitation (IP) of endogenous RNP complexes (RIP analysis) from whole-cell extracts was performed as described previously [57, 59, 60]. Briefly, cells were lysed in Protein Extraction Buffer (PEB) with 20 mM Tris-HCl at pH 7.5, 100 mM KCl, 5 mM MgCl_2_ and 0.5% NP-40 for 10 min on ice, and centrifuged at 10,000 × g for 15 min at 4°C. The supernatant was then incubated 1 h at 4°C with protein A-Sepharose beads coated with antibodies recognizing AUF1 (07-260, Millipore), or control IgG (Santa Cruz Biotechnology, Dallas, TX, USA ). After the beads were washed with NT2 buffer (50 mM Tris-HCl at pH 7.5, 150 mM NaCl, 1 mM MgCl_2_ and 0.05% NP-40), the complexes were incubated for 15 min at 37°C with 20 units of RNase-free DNase I. They were finally incubated for 15 min at 55°C with 0.1% SDS and 0.5 mg/ml Proteinase K, to remove remaining DNA and proteins, respectively. The RNA isolated from the IP materials was further assessed by Reverse transcription and quantitative PCR (RT-qPCR) analysis using the primers (Supplementary Table 1). Normalization of RIP results was carried out by quantifying, in parallel, the relative levels of housekeeping RNAs such as *GAPDH* mRNA in each IP sample. These abundant RNAs are non-specifically recovered during IP reactions.

### Western blot analysis

Whole-cell lysates, prepared in radioimmunoprecipitation assay buffer, were separated by sodium dodecyl sulfate-polyacrylamide gel electrophoresis and transferred onto nitrocellulose membranes (Invitrogen iBlot Stack). Anti-PGAM1 (sc-130334), anti-p16 (sc-1661), anti-p21 (sc-6246), anti-β-Actin (sc-47778) and anti-α-Tubulin (sc-5286) antibodies were purchased from Santa Cruz Biotechnology. Anti-MST1 (#3682), and anti-p-MST1 (#49332) were purchased from Cell Signaling Technology. Anti-PDP2 (ab133982) was purchased from Abcam. Anti-AUF1 (07-260) antibody was purchased from Millipore. A rabbit polyclonal phosphor-threonine 127 AUF1 antibody was produced by injection of a synthetic peptide (CEVVDCT(p)LKLDPIT) and affinity purification (Applied Biological Materials Inc., Richmond, Canada). The HRP-conjugated secondary antibodies were purchased from GE Healthcare (Dallas, TX, USA).

### RNA analysis

Trizol (Invitrogen) was used to extract total RNA and acidic phenol (Ambion) was used to extract RNA for RIP analysis. Reverse transcription (RT) was performed using random hexamers and reverse transcriptase (Maxima Reverse Transcriptase, Fermentas) and real-time, quantitative (q)PCR using gene-specific primers (Supplementary Table 1), and SYBR green master mix (Kapa Biosystems, Wilmington, MA, USA), using an Applied Biosystems (Waltham, MA, USA) 7300 instrument.

### Total RNA sequencing analysis

Total RNA was isolated using TRIzol, following the manufacturer’s instructions. For high-throughput RNA sequencing, we adhered to the standard Illumina protocol, employing the TruSeq Stranded Total RNA kit with Ribo-Zero (Part#15031048 Rev. E) and the NovaSeq6000 S4 for 150 bp paired-end sequencing. Analyses were conducted on two paired-end samples. The trimmed reads were mapped to the reference genome for MM9 using HISAT2. Following read mapping, StringTie was used for transcript assembly. The expression profile for each sample and transcript/gene was calculated as both read counts and Fragments per Kilobase of transcript per Million mapped reads.

### Isobaric Tags for relative and absolute quantitation (iTRAQ)

Samples were prepared using SDT lysis, followed by protein quantification utilizing the BCA method. The aliquoted samples were stored at −80°C. For each sample, 20 μg of protein was loaded onto a 12% SDS-PAGE gel and stained with Coomassie Brilliant Blue. Following FASP digestion, 100 μg of peptide from each sample was labeled according to the instructions provided by the iTRAQ Labelling Kit (, Concord, Canada). The labeled peptides from each group were pooled and fractionated using the Agilent 1260 Infinity II HPLC system. The samples were subsequently lyophilized and reconstituted in 0.1% formic acid before being divided into portions and separated using an Easy nLC system with nanoliter flow rates. The samples underwent chromatographic separation and were analyzed using a Q-Exactive mass spectrometer. The raw mass spectrometry data were identified and quantified with Mascot 2.5 and Proteome Discoverer 2.1 as described previously [58].

### Measurement of oxygen consumption rate by seahorse assay

Oxygen consumption rate (OCR) was measured using a Seahorse XFe96 Extracellular Flux Analyzer (Agilent, Santa Clara, CA, USA) in accordance with the manufacturer’s protocol. Cells were initially seeded into XF96 Cell Culture Microplates and incubated at 37°C for 24 hours. The culture medium was subsequently replaced with XF assay medium supplemented with 1 mM pyruvate, 2 mM glutamine, and 10 mM glucose. The cells were then incubated in a 37°C environment without CO_2_ for 1 hour. OCR was assessed through sequential injections of 1 μM oligomycin, 0.5 μM carbonyl cyanide 4-(trifluoromethoxy) phenylhydrazone, and 1 μM rotenone plus antimycin A, conducting a mitochondrial stress test using the XF Extracellular Flux Analyzer. Following the OCR assay, the cells were lysed, and protein concentration was measured to normalize the OCR data.

### Detection of glycolytic metabolites

Intracellular concentrations of 2-phosphoglycerate (2-PG), phosphoenolpyruvate (PEP), pyruvate and lactate were quantified using the glycolytic metabolite detection kits (BioVision, Milpitas, CA, USA) according to the manufacturer’s protocols.

### Auf1 and Mst1 KO mice

The Mst1 knockout mice were generated by crossing MST1^−/−^ Mst2^−/−^ mice with MST1^+/+^ mice obtained from Jackson Laboratory (Stock No: 017635), as previously described (29). Both Mst1^+/+^ and^−/−^ mice were utilized to assess the mRNA levels of *Pgam1* and *Pdp2*. Conditional knockout mice of Auf1 was generated by Cyagen Biosciences (Santa Clara, CA, USA).

### Senescence-associated (SA)-β-gal activity

The assessment of SA-β-gal activity was conducted in accordance with the manufacturer’s protocol (Cell Signaling Technology, Danvers, MA, USA). Briefly, fibroblasts were washed twice with 1× phosphate-buffered saline (PBS), fixed for 15 minutes at 25°C, and subsequently stained in a freshly prepared solution at pH 6.0. Images were acquired using a Nikon Digital Sight camera system attached to a Nikon Eclipse TS100 microscope. SA-β-gal-positive cells were quantified utilizing ImageJ, and the percentages of stained cells were calculated across three distinct fields [16].

### Quantification and statistical analysis

Data are expressed as the mean +/− S.D. of the values from at least three independent experiments performed, as indicated in the corresponding figures legends. The numbers of biological replicates, and what they represent, are indicated in each figure legend. Two-tailed Student’s *t*-tests were used for single comparison.

### Data and code availability

Data supporting the findings of this study are available upon request.

## Supplementary Materials

Supplementary Table 1
