# Supplementary material for: RNA-binding protein AUF1 suppresses cellular senescence and glycolysis by targeting PDP2 and PGAM1 mRNAs
Source: Aging (Albany NY). 2025 Jul 24;17(7):1746–61. doi: 10.18632/aging.206286 (PMC12339029; doi:10.18632/aging.206286)
Supplement: Supplementary Table 1 [file aging-17-206286-s001.pdf]

SUPPLEMENTARY TABLE

Supplementary Table 1. Primer sequences.

Name	Species	Sequence
GAPDH F	human	AGCCACATCGCTCAGACAC
GAPDH R	human	GCCCAATACGACCAAATCC
Gapdh F	mouse	AGGTCGGTGTGAACGGATTTG
Gapdh R	mouse	TGTAGACCATGTAGTTGAGGTCA
PGAM1 F	human	GCT CCT ATG ATG TCC CAC CA
PGAM1 R	human	ACA CGT TTC CCC TCC TTG AT
Pgam1 F	mouse	AGC GAC ACT ATG GCG GTC T
Pgam1 R	mouse	TGG GAC ATC ATA AGA TCG TCT CC
PDP2 F	human	GGTAGACGCTTATACTCCAGGT
PDP2 R	human	CACATGGGGAACTGTTTAGGG
Pdp2 F	mouse	TGATTTCAACAACGGAGTACCAA
Pdp2 R	mouse	CGCCATGTAGTAGAAAAGCCTCT
